# Fixed Oil from *Caryocar coriaceum*: Chemical Composition, Nutritional Relevance, Biological Activities, and Current Translational Challenges—A Scoping Review

**DOI:** 10.3390/foods15122185

**Published:** 2026-06-17

**Authors:** Joice Barbosa do Nascimento, Natália Kelly Gomes de Carvalho, José Galberto Martins da Costa

**Affiliations:** 1 Postgraduate Program in Biological Chemistry, Natural Products Research Laboratory, Department of Biological Chemistry, Regional University of Cariri, Rua Coronel Antônio Luíz, 1161—Pimenta, Crato 63105-010, Ceará, Brazil; joicenascimento2010@live.com; 2 Northeast Biotechnology Network—RENORBIO, State University of Ceará, Av. Dr. Silas Munguba, 1700—Campus do Itaperi, Fortaleza 60714-903, Ceará, Brazil; nataliakellygc@gmail.com

**Keywords:** functional lipids, nutraceuticals, bioactive compounds, food biotechnology, preclinical studies

## Abstract

*Caryocar coriaceum* Wittm. (Caryocaraceae) is a native Brazilian species predominantly distributed in Cerrado areas and transitional regions with the Caatinga in Northeastern Brazil, whose fruits exhibit significant nutritional, technological, and biofunctional potential. This review systematizes and critically analyzes the available scientific evidence regarding the fixed oil extracted from its fruits, addressing extraction methods, chemical composition, physicochemical parameters, nutritional value, technological applications, and the main bioactivities described in experimental models. Chromatographic and bromatological studies demonstrate that the oil presents a lipid profile characterized by the predominance of monounsaturated and saturated fatty acids, especially oleic acid and palmitic acid, in addition to the presence of carotenoids, phenolic compounds, and other bioactive lipophilic constituents. Available preclinical evidence indicates antioxidant, anti-inflammatory, wound-healing, gastroprotective, respiratory, anticonvulsant, and microbial resistance-modulating properties, suggesting potential applications in the food, pharmaceutical, cosmetic, and biotechnological fields. From the perspective of Food Science, the oil demonstrates characteristics compatible with lipid matrices of functional interest, although aspects related to oxidative stability, compositional standardization, sensory acceptability, and industrial scale-up remain insufficiently explored. Additionally, important limitations persist regarding the scarcity of systematic toxicological studies, the absence of clinical trials in humans, and the limited elucidation of the molecular mechanisms involved in the observed bioactivities. Although *C. coriaceum* presents promising biotechnological potential, its translational application still depends on further multidisciplinary studies integrating chemical standardization, toxicological safety, and technological development.

## 1. Introduction

*Caryocar coriaceum* Wittm. (Caryocaraceae) is a native Brazilian tree species distributed mainly in Cerrado areas and transitional zones with the Caatinga. The species has attracted increasing scientific interest due to its nutritional potential, economic importance, and traditional use by local communities in Northeastern Brazil [1].

Popularly known as “pequi” or “pequizeiro,” the species produces fruits with a fleshy, oily, and intensely pigmented pulp, widely used both in regional cuisine and in the production of fixed oil employed for artisanal and medicinal purposes [1,2,3].

Phytochemical and bromatological studies demonstrate that the fixed oil of *C. coriaceum* presents a composition rich in monounsaturated and saturated fatty acids, predominantly oleic acid and palmitic acid, in addition to the presence of carotenoids, phenolic compounds, tocopherols, and other lipophilic micronutrients capable of conferring nutritional and functional relevance to this plant matrix [4,5].

Over the last few decades, the search for new plant sources of functional lipids has gained considerable attention internationally, driven by the need for natural ingredients capable of combining technological stability, nutritional quality, and health-promoting potential. In this context, underexplored tropical species represent a strategic reservoir of lipid matrices with distinctive characteristics, among which *C. coriaceum* stands out due to its fatty acid profile and the presence of bioactive micronutrients, increasing scientific interest in its potential application as a functional ingredient, nutraceutical, and biotechnological resource [4,6].

Beyond its nutritional relevance, the traditional use of *C. coriaceum* oil has recurrently been associated with different empirical therapeutic purposes, including the treatment of inflammatory processes, respiratory disorders, skin lesions, gastric discomfort, and muscle pain [7,8,9]. Such popular use has motivated growing scientific interest in its pharmacological, nutritional, and technological investigation, resulting in a progressive increase in the number of studies focused on the chemical characterization of the oil and the evaluation of its experimental bioactivities in in vitro and in vivo models [2,4].

Despite this progress, the accumulated knowledge regarding *C. coriaceum* oil remains fragmented. The available evidence is dispersed among studies addressing chemical composition, physicochemical analyses, preclinical assays, and isolated technological applications, with a scarcity of integrative analyses capable of correlating chemical profile, food functionality, and mechanisms of biological action.

Furthermore, important limitations persist regarding the standardization of extraction methods, compositional reproducibility, identification of the constituents directly responsible for the observed bioactivities, and translational validation of the reported effects. This scenario justifies the need for a systematized critical review capable of gathering, organizing, and interpreting the currently available body of evidence.

In light of this context, the present review was developed with the purpose of systematizing and critically analyzing the available scientific evidence regarding the fixed oil of *C. coriaceum*, encompassing extraction methods, chemical profile, quality parameters, nutritional value, technological applications, and the main bioactivities described in experimental models. Additionally, this review sought to discuss the methodological limitations of the literature, the remaining gaps, and future perspectives for its consolidation as a functional ingredient, nutraceutical, or biotechnological resource.

The methodological approach followed the recommendations of the PRISMA-ScR guidelines for scoping reviews. Systematic searches were conducted in the ScienceDirect, PubMed^®^, SciELO, and Web of Science™ databases, covering studies published between 2006 and 2026, in English and Portuguese. The search strategy was adapted according to the indexing system and search syntax of each database.

The descriptors and Boolean operators used included combinations of the following terms in English and Portuguese: “*Caryocar coriaceum*”, “*Caryocar coriaceum* AND oil”, “*Caryocar coriaceum* AND fixed oil”, “pequi oil”, “fixed oil”, “fatty acids”, “bioactive compounds”, “chemical composition”, and “biological activity”, as well as their Portuguese equivalents “óleo”, “óleo fixo”, “óleo de pequi”, “ácidos graxos”, “compostos bioativos”, “composição química”, and “atividade biológica”. In PubMed^®^, searches were primarily performed using Title/Abstract fields, whereas in ScienceDirect and Web of Science™ searches included title, abstract, and keywords. In SciELO, searches were conducted using all indexed fields due to platform limitations.

The exact search strings used in each database are described below, and all searches were performed using equivalent strings in both English and Portuguese.

PubMed^®^: (“*Caryocar coriaceum*”[Title/Abstract]) AND (“oil”[Title/Abstract] OR “fixed oil”[Title/Abstract] OR “pequi oil”[Title/Abstract] OR “fatty acids”[Title/Abstract] OR “bioactive compounds”[Title/Abstract] OR “chemical composition”[Title/Abstract] OR “biological activity”[Title/Abstract]).

ScienceDirect: Title-Abstr-Key (“*Caryocar coriaceum*” AND (“oil” OR “fixed oil” OR “pequi oil” OR “fatty acids” OR “bioactive compounds” OR “chemical composition” OR “biological activity”)).

Web of Science™: Title-Abstr-Key (“*Caryocar coriaceum*” AND (“oil” OR “fixed oil” OR “pequi oil” OR “fatty acids” OR “bioactive compounds” OR “chemical composition” OR “biological activity”)).

SciELO: (“*Caryocar coriaceum*”) AND (“oil” OR “fixed oil” OR “pequi oil” OR “fatty acids” OR “bioactive compounds” OR “chemical composition” OR “biological activity”).

The eligibility process was conducted in sequential stages through the evaluation of titles, abstracts, and full texts. Studies were included when they presented original and consistent experimental data related to the extraction, physicochemical characterization, fatty acid composition, nutritional aspects, technological applications, or biological activities of *C. coriaceum* fixed oil. Review articles, duplicated records, studies unrelated to the fixed oil, publications lacking methodological detail, abstracts without full text availability, and studies outside the scope of the review were excluded.

**Figure 1:**
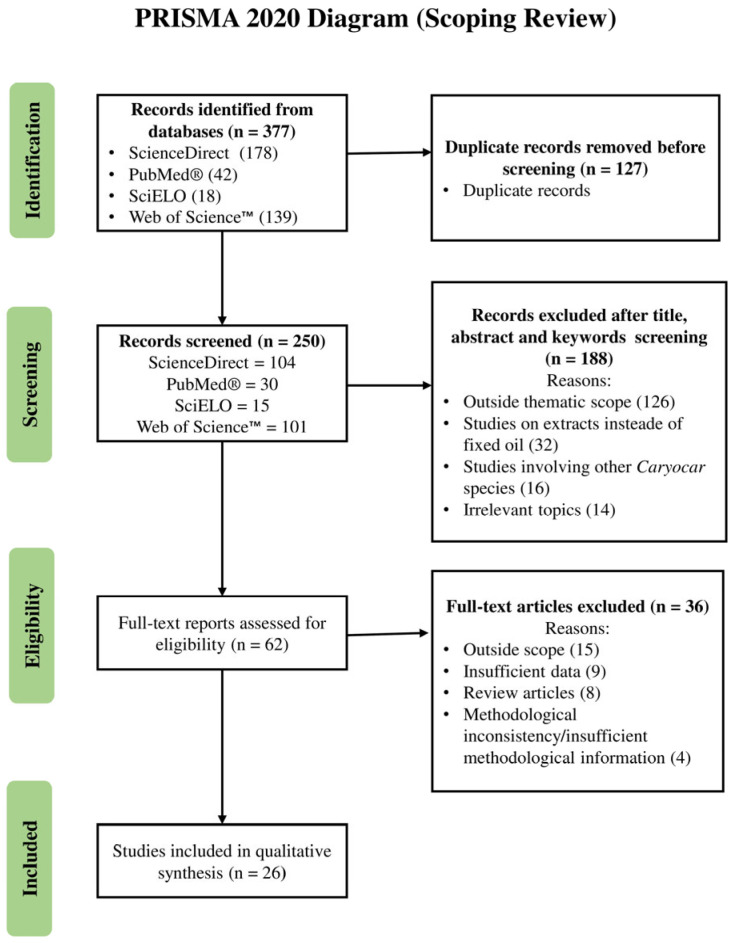
Flowchart of the process of identification, screening, and inclusion of studies, according to the PRISMA-ScR guidelines. Source: prepared by the authors (2026).

The study’s selection process was summarized using a PRISMA 2020 flow diagram. As illustrated in Figure 1, the initial search identified 377 records. After duplicate removal, title, abstract and keywords screening, eligibility assessment of full texts, and exclusion of studies due to methodological or thematic inadequacy, 26 studies were considered eligible for qualitative synthesis in the present review.

## 2. Botanical, Ecological, and Ethnobotanical Aspects

*C. coriaceum* occurs predominantly in Northeastern Brazil, especially in the states of Ceará, Pernambuco, and Piauí, where environmental conditions favor its development and natural occurrence. In the Chapada do Araripe region, the species plays an important ecological and socioeconomic role for traditional and extractivist communities, being widely associated with the food, medicinal, and commercial use of its fruits and derived products [2,3,10]. Its occurrence in transitional areas between the Cerrado and Caatinga contributes to its ecological adaptability and regional importance.

The species presents a robust arboreal habit and globose fruits characterized by fleshy, oily, and intensely pigmented pulp surrounding a spiny endocarp containing the almond. Although detailed morphological aspects are relevant from a taxonomic perspective, from a technological standpoint the main fraction of interest lies precisely in the pulp, which is responsible for concentrating most of the fixed oil used in culinary and medicinal applications [7,11].

From a morphoecological perspective, *C. coriaceum* presents characteristics compatible with species resistant to hydric seasonality, including coriaceous leaves, robust vegetative architecture, and adaptation to acidic soils with low fertility, factors that favor its persistence in environments subjected to prolonged drought periods. These adaptations partially explain the consolidation of the species as a widely accessible non-timber forest resource in semi-arid ecosystems, favoring its extractive exploitation by rural populations [2,10].

From an ethnobotanical perspective, pequi occupies a prominent position in regional food culture, being used in the preparation of traditional dishes, preserves, flours, and oily products for culinary use [12]. Simultaneously, the oil extracted from the pulp and, to a lesser extent, from the almond, is widely employed in folk medicine as an empirical therapeutic resource for the management of inflammatory conditions, respiratory disorders, muscle pain, burns, skin lesions, and gastric discomfort, reflecting a historical accumulation of traditional knowledge associated with the species [2,8,9].

In addition to its cultural value, the extractive chain of *C. coriaceum* presents considerable socioeconomic importance for rural populations and traditional communities in the Northeastern semi-arid region, especially during the fruiting season, when the collection and commercialization of fruits and derived products represent a complementary source of income. This scenario reinforces not only the conservationist interest but also the technological and industrial interest in the species, since the rational exploitation of its products may simultaneously contribute to the valorization of regional biodiversity, local income generation, and the development of higher value-added products [6,10].

Taken together, the botanical, ecological, and ethnobotanical aspects of *C. coriaceum* demonstrate that the species not only plays a relevant role in semi-arid ecosystems, but also constitutes a natural resource of high socioeconomic and functional value. The integration between the extensive food and medicinal use, and the regional extractive availability supports the growing scientific valorization of the fixed oil as a matrix of interest for food, nutraceutical, and biotechnological applications.

## 3. Extraction Methods, Chemical Composition, Physicochemical Quality, and Nutritional Value

The extraction of fixed oil from *C. coriaceum* represents a critical step in defining its chemical quality, oxidative stability, and technological performance, since the extraction method directly influences yield, preservation of lipophilic compounds, and the extent of degradative processes [13,14,15].

As shown in Table 1, the literature describes artisanal, mechanical, and chemical approaches for recovering the oily fraction, including pulp cooking in water, pressing, and extraction using organic solvents such as hexane and petroleum ether. Table 1 not only compiles protocols but also demonstrates that the choice of extraction method constitutes a determining variable for the final quality of the oily matrix analyzed in different studies.

The comparison among these methods demonstrates that, although artisanal and mechanical procedures are more compatible with maintaining sustainable appeal and future food applications, they frequently present lower recovery efficiency; in contrast, solvent-based methods tend to maximize yield, but introduce issues related to safety, chemical residues, and possible degradation of thermosensitive constituents [6].

The chemical characterization of *C. coriaceum* oil constitutes the main basis for interpreting its nutritional, technological, and biological properties. For fatty acid profiling, the lipid fraction is commonly subjected to derivatization by transesterification, generally catalyzed by acidic or basic reagents (e.g., NaOH or KOH), to obtain free fatty acid methyl esters (FAMEs) suitable for gas chromatographic analysis. However, it is important to emphasize that this derivatization approach is mainly applicable to fatty acid analysis and may not adequately preserve other bioactive constituents, such as carotenoids and tocopherols, which can undergo thermal degradation or oxidative modifications during sample preparation and high-temperature chromatographic procedures. Therefore, complementary analytical approaches are necessary for a more comprehensive characterization of the bioactive composition of the oil [18].

In general, the studies compiled in Table 2 demonstrate the relative percentages of total fatty acids, composed predominantly of monounsaturated and saturated fatty acids, especially oleic acid and palmitic acid, in addition to smaller proportions of other compounds [4,5].

However, the critical interpretation of Table 2 reveals that the expressive percentage variation observed for the major fatty acids among different studies should not be interpreted merely as an analytical discrepancy, but mainly as a reflection of the influence of factors such as geographical origin, fruit maturation stage, analyzed botanical fraction, postharvest processing, and the extraction method employed.

To date, no studies have reported the occurrence of other classes of compounds in the fixed oil of *C. coriaceum*, revealing a significant gap in current knowledge. Nevertheless, studies on *Caryocar brasiliense*, a phylogenetically related species sharing notable botanical, ecological, and phytochemical similarities with *C. coriaceum*, have identified minor bioactive constituents such as tocopherols and phytosterols (Table 2). Furthermore, total phenolic contents of 113.01 ± 10.40 and 252.00 ± 31.80 mg GAE/100 g oil, together with total carotenoid contents of 118.42 ± 2.57 and 89.82 ± 18.09 μg/g, have been reported for oils obtained through manual extraction and cold pressing, respectively [19,20].

**Table 2:** Main chemical constituents and percentages relative to the total fatty acids of the fixed oil of *C. coriaceum* and *C. brasiliense*.

Compounds	Specie	Extraction Method	Chemical Class	Unit of Measurement	Pequi Fraction	Reference
*cis*-9-oleic acid	*C. coriaceum* *C. brasiliense*	Aqueous extraction	Unsaturated fatty acid	61.25%	Fruits	[5]
				62.61%	Fruits	[16]
		Extraction by hydraulic pressing		42.47%	Fruits	[17]
		Extraction with organic solvents		43.59%	Almonds	[6]
		Aqueous and enzymatic extraction		54.55%	Pulp	[15]
Palmitic acid	*C. Coriaceu* *C. brasiliense*	Aqueous extraction	Saturated fatty acid	32.40%	Fruits	[5]
				33.40%	Fruits	[16]
		Extraction by hydraulic pressing		39.49%	Fruits	[17]
		Extraction with organic solvents		43.76%	Almonds	[6]
		Aqueous and enzymatic extraction		43.61%	Pulp	[15]
Linolenic acid	*C. coriaceum* *C. brasiliense*	Extraction by hydraulic pressing	Polyunsaturated fatty acid	10.17%	Fruits	[17]
		Aqueous and enzymatic extraction		0.18%	Pulp	[15]
Stearic acid	*C. coriaceum* *C. brasiliense*	Aqueous extraction	Saturated fatty acid	1.74%	Fruits	[5]
		Extraction by hydraulic pressing		1.63%	Fruits	[17]
		Extraction with organic solvents		2.54%	Almonds	[6]
		Aqueous and enzymatic extraction		0.66%	Pulp	[15]
*gamma*-linolenic acid	*C. coriaceum* *C. brasiliense*		Polyunsaturated fatty acid	2.23%	Fruits	[5]
*cis*-vaccenic acid	*C. coriaceum* *C. brasiliense*	Extraction with organic solvents	Unsaturated fatty acid	1.38%	Almonds	[6]
Palmitoleic acid	*C. coriaceum* *C. brasiliense*	aqueous extraction	Unsaturated fatty acid	0.41%	Fruits	[5]
		Extraction with organic solvents		1.23%	Almonds	[6]
*cis*-11-eicosenoic acid	*C. coriaceum* *C. brasiliense*	Aqueous extraction	Unsaturated fatty acid	0.73%	Fruits	[5]
Linoleic acid	*C. coriaceum* *C. brasiliense*		Polyunsaturated fatty acid	0.84%	Pulp	[10]
		Aqueous extraction		0.39%	Fruits	[5]
Myristic acid	*C. coriaceum* *C. brasiliense*	Aqueous extraction	Saturated fatty acid	0.06%	Fruits	[5]
		Aqueous and enzymatic extraction		0.22%	Fruits	[15]
		Extraction with organic solvents		0.46%	Almonds	[6]
*α*-tocopherol	*C. brasiliense*	Manual extraction	Tocopherols	86.00 ± 2.57 mg/kg	Almonds	[20]
		Cold pressing	Tocopherols	91.49 ± 4.01 mg/kg	Almonds	[20]
*γ*-tocopherol	*C. brasiliense*	Manual extraction	Tocopherols	68.28 ± 5.33 mg/kg	Almonds	[20]
		Cold pressing	Tocopherols	63.82 ± 8.82 mg/kg	Almonds	[20]
Campesterol	*C. brasiliense*	Manual extraction	Fitosterols	43.58 ± 2.56 mg/kg	Almonds	[20]
		Cold pressing	Fitosterols	42.82 ± 1.77 mg/kg	Almonds	[20]
Stigmasterol	*C. brasiliense*	Manual extraction	Fitosterols	521.65 ± 15.31 mg/kg	Almonds	[20]
		Cold pressing	Fitosterols	527.30 ± 36.74 mg/kg	Almonds	[20]
*β*-sitosterol	*C. brasiliense*	Manual extraction	Fitosterols	228.09 ± 13.62 mg/kg	Almonds	[20]
		Cold pressing	Fitosterols	238.50 ± 12.90 mg/kg	Almonds	[20]

**Figure 2:**
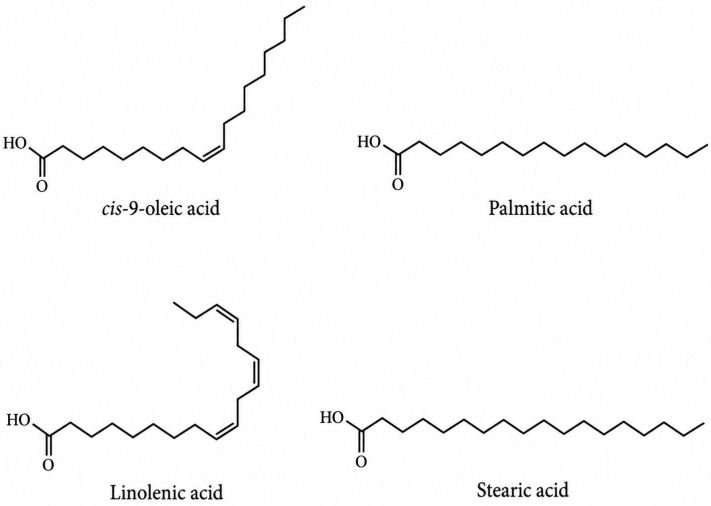
Structural representation of the main free fatty acids identified in the fixed oil of *C. coriaceum.* Source: prepared by the authors (2026).

Such compositional variation has a direct implication for oxidative stability, rheological behavior, and possibly the magnitude of the biological effects reported for the oil, representing a relevant challenge for its future industrial standardization and for more precise comparisons among independent investigations. Thus, Table 2 plays a central role by simultaneously evidencing the predominance of the monounsaturated profile and the compositional heterogeneity still existing in the literature [5,6]. The chemical structures of the main constituents commonly identified in *C. coriaceum* oil are represented in Figure 2.

From a bromatological perspective, the data compiled in Table 3 confirm that *C. coriaceum* constitutes a plant matrix with a high lipid content and potentially high energy density, particularly in the pulp and kernel fractions. In addition to the substantial lipid levels, moderate amounts of proteins, dietary fibers, and ash have been reported, along with the presence of micronutrients and other constituents associated with antioxidant activity.

A comparative analysis of the data presented in Table 3 reveals considerable variability in the proximate composition of the species. The differences observed among studies may be attributed to genetic, environmental, and methodological factors, including cultivation conditions, fruit maturity stage, sample storage conditions, and the analytical procedures employed [21,22]. Therefore, the reported values should be interpreted as representative ranges reflecting the variability documented in the literature.

**Table 3:** Centesimal composition of the fixed oil of *C. coriaceum* from the pulp and kernel fractions.

Moisture	Ash	Proteins	Lipids	Carbohydrates	Material	Reference
(%)						
0.65–61.56	0.28–3.18	1.60–29.24	18.95–70.16	3.60–59.93	Pulp	[6,10,15,17,23]
47.62–53.21	2.26–3.05	23.89–33.84	44.89–55.14	14.62–26.91	Almonds	[23]

Despite this variability, the available evidence supports the characterization of the fruit as a nutritionally relevant raw material. Furthermore, the high moisture content observed in freshly harvested fruits may promote hydrolytic reactions, enzymatic activity, and microbial deterioration, thereby compromising the stability of the raw material when processing is delayed. This scenario highlights the importance of drying or oil extraction shortly after harvest to preserve the quality of bioactive constituents and lipids. Collectively, these characteristics underscore the potential of *C. coriaceum* as a promising raw material not only for oil production but also for its integral utilization in value-added food formulations [5].

Regarding physicochemical quality parameters, Table 4 summarizes the acid, peroxide, saponification, and iodine values, which are widely used to evaluate lipid integrity, oxidative stability, and the degree of unsaturation of the oil matrix.

Although some results suggest a favorable preservation profile, the comparative analysis of the data reveals considerable variability among batches and analytical methodologies, which limits broad generalizations regarding the intrinsic stability of the oil. In practice, its stability depends not only on the fatty acid composition, particularly the degree of unsaturation, but also on extraction and storage conditions, including residual moisture content, exposure to light, oxygen, and temperature, as well as the presence of minor constituents with antioxidant or pro-oxidant activity.

In this context, Martins et al. [10], using a combination of solvent extraction and mechanical pressing to obtain pequi pulp oil, reported a higher iodine value (25.98 mg), indicating a greater degree of lipid unsaturation, in addition to an increased peroxide value (79.5 mg), which demonstrated a higher extent of lipid oxidation attributed to the heating of the miscella during the solvent extraction process.

Mariano et al. [15] obtained pequi pulp oil through aqueous and enzymatic extraction methods. The authors observed that the acid value of the oil obtained by aqueous extraction (2.85 mg KOH/g) was similar to that of commercial oil (2.48 mg KOH/g). According to the authors, these values were associated with the high processing temperature (80 °C), which favored lipid hydrolysis reactions. Furthermore, enzymatic treatments promoted greater oxidation of unsaturated fatty acids, resulting in a reduction in the lipid content of the oil.

Lima et al. [17], when extracting pequi almond oil by hydraulic pressing, demonstrated that the applied pressure and moisture content significantly influenced extraction yield. Additionally, factors such as cultivar, geographic origin, maturation stage, and postharvest conditions constitute determining variables affecting fatty acid composition and the physicochemical properties of the oil.

Therefore, the results presented in Table 4 reinforce the need for controlled studies on oxidative stability and shelf life to achieve a more robust characterization of the technological profile and quality of pequi oil.

From the perspective of Food Science, the integrated set of Table 2, Table 3 and Table 4 demonstrates that *C. coriaceum* oil attracts interest not only because of its favorable lipid profile, but also due to the possibility of incorporation into food matrices aimed at the development of functional products. The high oleic acid content, associated with carotenoids, phenolic compounds, and other minor lipophilic constituents, may contribute to nutritional enrichment, enhancement of antioxidant capacity, and improvement of the biological value of different formulations.

Preliminary studies involving its application in dairy products and enriched food systems indicate initial technological feasibility, suggesting potential similar to that observed in functional oils already established in the market, such as olive oil and avocado oil. Nevertheless, parameters such as sensory acceptability, behavior during thermal processing, pilot-scale microencapsulation, and shelf life remain insufficiently explored [5,6].

Taken together, Table 1, Table 2, Table 3 and Table 4 demonstrate that *C. coriaceum* oil presents a chemical, bromatological, and technological profile compatible with lipid matrices of functional interest, while simultaneously evidencing the need for greater methodological standardization for its industrial and scientific consolidation. This compositional and qualitative basis supports the growing number of investigations focused on the evaluation of its experimental bioactivities, discussed below.

## 4. Preclinical Evidence of the Bioactivities of *C. coriaceum* Oil

Biological evidence related to *C. coriaceum* oil has expanded considerably in recent years, especially in preclinical models designed to evaluate its ability to modulate oxidative stress, inflammation, tissue injury, and metabolic disorders. Although short-term experimental assays still predominate, the set of available results allows the identification of a bioactive lipid matrix of pharmacological and nutritional interest.

As summarized in Table 5, the main investigations are concentrated in seven recurrent biological fronts, involving antioxidant/anticonvulsant, anti-inflammatory, respiratory, wound-healing, gastroprotective, metabolic, and antimicrobial modulatory activities.

Within the scope of redox homeostasis and neuroprotection, the anticonvulsant and antioxidant potential of the fixed oil of *C. coriaceum* has been associated with its ability to attenuate oxidative stress and preserve cellular stability in models of neurochemical injury. In mice subjected to pentylenetetrazol-induced seizures, oral administration of 100 mg/kg significantly increased the latency to myoclonic spasms and tonic–clonic seizures, without behavioral alterations compatible with acute toxicity. These findings suggest a neuroprotective effect possibly related to the modulation of oxidative pathways and the maintenance of neuronal redox homeostasis, although the specific molecular targets remain insufficiently explored [4].

In addition to the effects related to redox protection, the literature demonstrates that this lipid matrix exerts relevant modulation on acute inflammatory events. In different murine models of topical or systemic edema and inflammation, consistent reductions in cellular infiltrate, edema, and enzymatic parameters associated with the inflammatory cascade were observed, providing scientific support for the traditional ethnomedicinal use of the oil in peripheral inflammatory processes [2]. However, it is important to emphasize that such effects should not be attributed exclusively to oleic acid, but possibly to the high content of monounsaturated fatty acids in synergy with carotenoids, phenolic compounds, and other minor lipophilic constituents that have not yet been completely individualized.

The impact of these anti-inflammatory properties becomes particularly evident in lung tissue. Models involving exposure to smoke and other inhaled pollutants demonstrated that prior administration of the oil is capable of preserving respiratory mechanics parameters, reducing inflammatory cell infiltration, and limiting histomorphological damage to the pulmonary parenchyma. The recurrence of this effect in distinct experimental systems strengthens the hypothesis of a convergent cytoprotective mechanism based on attenuation of oxidative stress and the local inflammatory response, although without definitive molecular elucidation.

In parallel with the protection of internal tissues, the ability of the oil to modulate inflammation and oxidative stress is also reflected in peripheral repair processes. Wound-healing activity constitutes one of the most consistently reported bioactivities in the literature, with favorable evidence in different models of skin and tendon injury.

In general, studies point to acceleration of wound contraction, increased reepithelialization, greater collagen deposition, and improved organization of regenerating tissue, suggesting a biologically relevant reparative response. Nevertheless, the exact contribution of each chemical subfraction and the cellular mechanisms involved in angiogenesis, fibroblast migration, and tissue remodeling remain insufficiently elucidated [2,5].

Gastric protection represents another promising pharmacological front for *C. coriaceum* oil. In acute models of lesions induced by ethanol and nonsteroidal anti-inflammatory drugs, significant reduction in the ulcerated area and preservation of gastric mucosal structure were observed, suggesting the involvement of multiple cytoprotective pathways. Although the results are favorable, the relative contribution among prostaglandin-dependent mechanisms, nitric oxide, KATP channels, and antioxidant action remains undefined, requiring more in-depth pharmacological investigations before any translational extrapolation [4].

In the metabolic field, the available findings are more heterogeneous. Some studies report partial reduction in cholesterol and triglycerides and slight improvement in biochemical parameters in hyperlipidic diet models, whereas others do not demonstrate consistent effects on weight gain or global metabolic remodeling. This apparent inconsistency suggests that the hypolipidemic effects of the oil should not be interpreted as linear or universal, but rather as dependent on the administered dose, duration of intervention, pathophysiological context, and the compositional variability of the oil used in each assay [6].

Finally, the antibacterial activity of the fixed oil of *C. coriaceum* has been investigated mainly in in vitro assays, in which its direct action on microbial growth tends to be moderate. More consistent results have been reported when the oil is evaluated in association with conventional antibiotics, suggesting a more relevant role as a bacterial resistance modulator rather than as an isolated antimicrobial agent. From this perspective, the literature points to possible interference with membrane permeability or bacterial efflux mechanisms, a fundamental distinction to avoid overestimation of its antimicrobial potential [5].

Taken together, the different biological activities attributed to the fixed oil of *C. coriaceum* appear to converge toward a central mechanistic axis based on the modulation of oxidative stress, attenuation of inflammatory responses, and preservation of cellular structures against different chemical and physical insults.

Although Table 5 demonstrates promising biological effects in different experimental models, the contribution of individual constituents remains unclear. To date, no studies have investigated isolated compounds from *C. coriaceum* oil, and systematic fractionation approaches are still scarce. Consequently, the specific roles and possible synergistic interactions among fatty acids, carotenoids, phenolic compounds, and other minor lipophilic constituents remain poorly understood, limiting a more robust mechanistic interpretation of the reported bioactivities.

## 5. Biotechnological Potential and Associated Patents

Beyond the chemical, nutritional, and pharmacological evidence, the fixed oil and other fractions derived from *C. coriaceum* have attracted interest in the field of technological innovation, although still incipiently. The patent survey conducted in databases such as the World Intellectual Property Organization, the Brazilian National Institute of Industrial Property, and the World Intellectual Property Organization identified four records directly related to applications of the species in the food, pharmaceutical, cosmetic, and sanitizing industries, with deposits concentrated between 2018 and 2023.

Although still numerically modest, these patent deposits reveal an important change in the scientific status of *C. coriaceum*, indicating the gradual transition from a species traditionally exploited in a regional context to a plant platform of biotechnological interest. It is noteworthy that the identified patents are not restricted to conventional food use, but encompass technologies ranging from pulp drying and stabilization to nanostructured delivery systems and multifunctional pharmaceutical and sanitary formulations, evidencing progressive diversification of innovation routes.

In the food sector, patents BR 102018077530 and BR 102018016170 focus on obtaining powdered derivatives from the pulp of *C. coriaceum*, including the production of functional seasoning and the development of dehydrated pulp through convective drying. In both cases, there is a technological concern regarding maintenance of the typical sensory characteristics of pequi, nutritional preservation, and expansion of storage stability, demonstrating an attempt to convert a seasonal and perishable product into a food ingredient with longer shelf life and greater industrial added value. These records directly correlate with the findings presented in Table 3 and Table 4, which demonstrate both the nutritional value of the matrix and the need for conservation and processing strategies to maintain its functional integrity.

From the pharmaceutical perspective, patent BR 102019024042 significantly broadens the translational horizon of the species by describing a nanoformulation containing *C. coriaceum* oil for topical use in knee osteoarthritis. The use of polymeric nanocapsules to increase the stability, solubility, and efficacy of the oil demonstrates that the species is beginning to be explored not only as a raw material, but also as an active component in advanced delivery systems, bringing academic research closer to more sophisticated clinical and pharmaceutical applications.

Even more comprehensively, patent BR 102023027766 describes a multifunctional nanoemulsion associating *C. coriaceum* oil with the essential oil of *Lippia sidoides*, with pharmaceutical, cosmetic, and sanitizing applications. The highlight of this deposit lies in the use of nanostructuring to enhance larvicidal, repellent, anti-inflammatory, and wound-healing properties, in addition to proposing a natural and environmentally safer alternative to synthetic formulations. This record demonstrates that the biotechnological exploitation of the species is beginning to surpass simple nutritional use, reaching integrated biofunctionalization strategies and hybrid systems of greater technological complexity.

Taken together, the patent panorama reveals that, although the number of deposits is still reduced and predominantly concentrated at the national level, *C. coriaceum* already presents concrete signs of translational maturation. The predominance of applications directed toward food stabilization and bioactive nanoformulations suggests that the main innovation axes currently associated with the species are centered on shelf-life extension, increased bioavailability, and potentiation of biological effects previously demonstrated in experimental studies. Thus, the patent scenario reinforces that interest in *C. coriaceum* has already surpassed the descriptive academic sphere, progressively advancing toward intellectual protection and the development of products with greater technological value.

## 6. Current Limitations and Future Perspectives

Despite the growing number of investigations on the fixed oil of *C. coriaceum*, its scientific and technological consolidation still faces substantial limitations. First, there is marked heterogeneity among studies regarding botanical origin, seasonality, fruit maturation stage, extraction methods, and postharvest processing, factors that directly influence the lipid profile and the reproducibility of biological results. As evidenced in Table 1 and Table 2, both extraction protocols and fatty acid composition present considerable variation among independent investigations, which hinders direct comparisons and represents one of the main challenges for future proposals of chemical standardization and industrial scale-up.

Although the available preclinical evidence demonstrates that *C. coriaceum* oil has a promising effect, important gaps remain insufficiently explored. Most investigations are based on acute preclinical or in vitro models, predominantly involving rodents or alternative experimental systems, with limited exploration of chronic exposure, pharmacokinetics, bioavailability, long-term toxicological safety, and translational applicability to humans. In addition, considerable heterogeneity among studies regarding extraction methods, oil composition, doses, treatment duration, and experimental protocols compromises direct comparisons and hinders the establishment of standardized biological responses.

Another major limitation concerns the insufficient elucidation of the molecular mechanisms underlying the reported bioactivities. Although anti-inflammatory and antioxidant effects are recurrently associated with modulation of oxidative stress and inflammatory pathways, few studies have directly investigated mediators such as cytokines, COX/LOX enzymes, reactive oxygen species, nitric oxide, and antioxidant enzymes through mechanistic approaches. Likewise, the specific bioactive compounds responsible for the observed effects remain largely unexplored. To date, no studies have systematically evaluated isolated compounds from *C. coriaceum* oil, making it difficult to determine the individual contribution of fatty acids, carotenoids, phenolic compounds, and other minor lipophilic constituents, as well as their possible synergistic interactions.

Therefore, future investigations should prioritize systematic fractionation approaches, isolation and characterization of active compounds, omics-based analyses, standardized experimental protocols, chronic toxicity studies, and controlled clinical trials. These advances will be essential to clarify the mechanistic basis of the reported effects and to support the consolidation of *C. coriaceum* oil as a functional ingredient, nutraceutical, or biotechnological resource with validated safety, efficacy, and industrial applicability.

One of the most relevant bottlenecks for validating the oil as a functional food ingredient or nutraceutical input lies in the scarcity of systematic toxicological data. To date, there is insufficient evidence regarding long-term oral toxicity, genotoxicity, reproductive effects, metabolic interactions, or the establishment of safe intake limits, representing a critical gap for any projection of regular human use. This limitation becomes even more significant considering the growing proposal for incorporating the oil into food formulations and nutraceutical systems intended for continuous consumption.

Regarding the field of Food Science, parameters such as shelf-life stability, sensory acceptability, behavior during thermal processing, pilot-scale microencapsulation, and economic feasibility of industrial production remain insufficiently explored. Although Table 3 and Table 4 indicate favorable nutritional and physicochemical attributes, the literature still lacks multidisciplinary studies capable of connecting chemical standardization, biological evaluation, toxicological safety, and applied technological development. In this sense, future investigations should prioritize integrated approaches capable of converting the current experimental potential into effective industrial and translational applicability.

## 7. Conclusions

In summary, the fixed oil of *C. coriaceum* constitutes a lipid matrix of considerable nutritional, technological, and biotechnological interest, whose composition rich in monounsaturated fatty acids and lipophilic micronutrients supports consistent preclinical evidence in different experimental models. The integration of the data presented in Table 1, Table 2, Table 3, Table 4 and Table 5 demonstrates that the oil presents favorable conditions regarding composition, physicochemical quality, nutritional value, and biofunctionality, in addition to promising applicability as a value-added food ingredient.

The currently available studies indicate functional potential in antioxidant, anti-inflammatory, wound-healing, gastroprotective, respiratory, antimicrobial modulatory, and metabolic spheres, reinforcing the scientific relevance of a species traditionally exploited by communities of the Northeastern semi-arid region. Nevertheless, the transition of this empirically used input into a validated nutraceutical or functional resource still faces important barriers, especially with regard to chemical standardization, elucidation of molecular mechanisms of action, long-term toxicological evaluation, and the absence of clinical trials in humans.

Thus, the advancement of knowledge regarding *C. coriaceum* will depend on multidisciplinary approaches capable of connecting regional biodiversity, Food Science, experimental pharmacology, and translational medicine, allowing the potential currently supported by preclinical evidence to be progressively converted into technological innovation and safe large-scale application.

## Figures and Tables

**Table 1:** Extraction methods of fixed oil from *C. coriaceum*.

Extraction Method	Plant Material	Procedure	Yield	Characterization Technique	Reference
Artisanal extraction	Fruits	The fruits are placed in boiling water for 40 min, where the oil is separated from the pulp.	14.15%	GC/FID	[16]
Artisanal and mechanical extraction	Fruits	Heating at 45 °C followed by centrifugation at 4500 rpm for 15 min.		GC/FID	[5]
Solid–liquid extraction	Fruits	The material is placed in contact with ethyl acetate for 3 h (60 °C) in a Soxhlet apparatus, followed by decantation and solvent removal using a rotary evaporator.		GC/FID	[2]
Solid–liquid and mechanical extraction	Fruits	Material immersed in hexane for 7 days followed by solvent removal. The material is subsequently pressed and centrifuged.	59.10%	GC/FID	[10]
Mechanical extraction	Almonds	The material is conditioned in TNT60 bags in an oven for 15 min at 40 °C. Afterwards, samples are subjected to a stainless-steel cylinder and pressed by a piston until the test force is reached, remaining for 3 min.	75.00%	GC/FID	[17]

**Table 4:** Quality parameters reported in the literature for fixed oil of *C. coriaceum*.

		Index				Reference
Plant Part	Extraction Method	Acidity (mg KOH/g)	Peroxide (meq O_2_/kg)	Iodine (g I_2_ 100/g)	Saponification (mg KOH/g)	
Almond	Hydraulic pressing	0.17	1.22	54.13	209.65	[17]
Pulp	Combination of solvent extraction and mechanical pressing		25.98	79.5		[10]
Pulp	Aqueous extraction	2.85	1.16	52.03	180.13	[15]
Pulp	Aqueous extraction	1.35	3.15	74.80	184.23	[15]

**Table 5:** Summary of the biological activities of *C. coriaceum*.

Activity	Experimental Model	Dose/Concentration	Main Effects	Reference
Anticonvulsant	Mice	100 mg/kg	↑ seizure latency; no effect on duration	[4]
Antioxidant (in vitro)	DPPH	IC_50_ ≈ 10.21 mg/mL	Radical scavenging activity	[4]
Antioxidant (in vivo)	*D. melanogaster* + paraquat	1.5 or 10 mg/g	↓ ROS, ↓ lipid peroxidation, ↑ viability	[24]
Antioxidant (biotechnology)	Bovine semen	1.0–1.5 mL/L	↓ MDA; no functional improvement	[25]
Anti-inflammatory	Rats (ear edema)	in natura (100%)	~39% edema inhibition	[26]
Anti-inflammatory	Rats (paw edema)	500, 1000 and 2000 mg/kg	↓ edema, ↓ MPO	[3]
Anti-inflammatory	Mice (cutaneous edema)	13 mg/ear	Up to 49.7% inhibition	[2]
Respiratory	Rats (acute cigarette smoke exposure)	0.5 mL	Improved pulmonary function	[27]
Respiratory	Mice (diesel particles)	0.5 mL	↓ PMN, ↓ BCI	[28]
Respiratory	Mice (biomass smoke)	50 μL	↓ inflammatory cell infiltration	[29]
Wound-healing	Mice (cutaneous wound)	10–12% topical	Up to 96.5% wound contraction	[26]
Wound-healing	Rats (cutaneous wounds)	10% oil	Complete reepithelialization	[30]
Gastroprotective	Mice (ethanol- and aspirin-induced gastric ulcer)	200–400 mg/kg	~60–67% lesion reduction	[8]
Gastroprotective	Mice (ethanol-induced gastric ulcer)	200–400 mg/kg	~57–61% inhibition	[31]
Hypolipidemic	Rats	500–2000 mg/kg	↓ cholesterol (16%), ↓ TG (23%), ↑ HDL (71%)	[3]
Metabolic	Obese mice	100 mg/kg	No metabolic effect	[16]
Antibacterial	In vitro (microdilution)	MIC ≥ 813 µg/mL	Moderate activity	[9]
Antibacterial (synergy)	In vitro (microdilution)	MIC 512 mg/mL	Moderate activity, ↓ antibiotic MIC up to 99.8%	[2]
Antibacterial	In vitro (agar diffusion method)	15–13 mm and MICs of 1.25%	Greater activity against Gram-positive bacteria	[32]
Antibiofilme	In vitro (microdilution)	10 mg/mL	~70% biofilm inhibition	[33]
Larvicidal	*A. aegypti*	500 ppm	100% mortality	[34]

Note: ↑ indicates an increase; ↓ indicates a decrease.

## Data Availability

No new data were created or analyzed in this study. Data sharing is not applicable to this article.
